# *Staphylococcus aureus* strains associated with food poisoning outbreaks in France: comparison of different molecular typing methods, including MLVA

**DOI:** 10.3389/fmicb.2015.00882

**Published:** 2015-09-11

**Authors:** Sophie Roussel, Benjamin Felix, Noémie Vingadassalon, Joël Grout, Jacques-Antoine Hennekinne, Laurent Guillier, Anne Brisabois, Fréderic Auvray

**Affiliations:** Université Paris-Est, ANSES, Food Safety Laboratory, European Union Reference Laboratory for Coagulase Positive Staphylococci, Maisons-AlfortFrance

**Keywords:** food poisoning outbreaks, *Staphylococcus aureus*, MLVA, PFGE, spa-typing, enterotoxin genes, genetic diversity

## Abstract

Staphylococcal food poisoning outbreaks (SFPOs) are frequently reported in France. However, most of them remain unconfirmed, highlighting a need for a better characterization of isolated strains. Here we analyzed the genetic diversity of 112 *Staphylococcus aureus* strains isolated from 76 distinct SFPOs that occurred in France over the last 30 years. We used a recently developed multiple-locus variable-number tandem-repeat analysis (MLVA) protocol and compared this method with pulsed field gel electrophoresis (PFGE), *spa*-typing and carriage of genes (*se* genes) coding for 11 staphylococcal enterotoxins (i.e., SEA, SEB, SEC, SED, SEE, SEG, SEH, SEI, SEJ, SEP, SER). The strains known to have an epidemiological association with one another had identical MLVA types, PFGE profiles, spa-types or *se* gene carriage. MLVA, PFGE and *spa*-typing divided 103 epidemiologically unrelated strains into 84, 80, and 50 types respectively demonstrating the high genetic diversity of *S. aureus* strains involved in SFPOs. Each MLVA type shared by more than one strain corresponded to a single spa-type except for one MLVA type represented by four strains that showed two different-but closely related-spa-types. The 87 enterotoxigenic strains were distributed across 68 distinct MLVA types that correlated all with *se* gene carriage except for four MLVA types. The most frequent *se* gene detected was *sea*, followed by *seg* and *sei* and the most frequently associated *se* genes were *sea-seh* and *sea-sed-sej-ser*. The discriminatory ability of MLVA was similar to that of PFGE and higher than that of spa-typing. This MLVA protocol was found to be compatible with high throughput analysis, and was also faster and less labor-intensive than PFGE. MLVA holds promise as a suitable method for investigating SFPOs and tracking the source of contamination in food processing facilities in real time.

## Introduction

Staphylococcal food poisoning is one of the most common food-borne diseases worldwide (Yu et al., 2007; Kadariya et al., 2014). It results from the ingestion of SEs preformed in food and produced by enterotoxigenic strains of CPS (Argudin et al., 2010). SEs are frequent causes of food-borne outbreaks in Europe (EFSA-ECDC, 2013). Two types of SFPOs can be differentiated. Outbreaks for which the evidence implicating a particular food vehicle is strong, based on the assessment of all available data, are referred to as “strong-evidence SFPO,” whereas outbreaks for which no particular food vehicle is suspected or where the evidence implicating a particular food vehicle is weak are referred to as “weak-evidence SFPO” (EFSA, 2011).

Among the seven described species belonging to the CPS group, *Staphylococcus aureus* ssp. *aureus* is the main causative agent of SFPOs. To date, 21 SEs have been described: SEA to SElV all possess superantigenic activity whereas only a subset of SEs (i.e., SEA to SEI, SER, SES, and SET) are emetic (Ono et al., 2008). Out of the 21 SEs, 11 (i.e., SEA, SEB, SEC, SED, SEE, SEG, SEH, SEI, SEJ, SEP, SER) are suspected to cause SFPOs (Hennekinne et al., 2011).

Few data is available on the genetic diversity of the strains isolated from SFPOs. Among the molecular methods available, pulsed field gel electrophoresis (PFGE) and *Staphylococcus* protein A gene (*spa*) typing have been extremely helpful in short-term investigations and identification of SFPOs (Chiou et al., 2000; Shimizu et al., 2000; Wei and Chiou, 2002; Strommenger et al., 2006; Dyer et al., 2007; Hallin et al., 2007; Kerouanton et al., 2007; Kellermann et al., 2008; Ostyn et al., 2010; Wattinger et al., 2012; Chiang et al., 2014). Although PFGE is highly discriminatory, it remains a time-consuming and labor intensive method. It also requires highly skilled operators and there are no standardized reagents. Moreover, profile interpretation requires several subjective decisions, increasing the variability of the profiles and leading to possible uncertainty about profiles relatedness (Cookson et al., 1996; Murchan et al., 2003).The advantages of *spa*-typing are its excellent inter-laboratory reproducibility, the portability of the data and its flexible analysis throughput (Koreen et al., 2004; Aires-de-Sousa et al., 2006; Cookson et al., 2007). However, this method is less discriminatory than PFGE for the characterization of food isolates (Babouee et al., 2011).

Identification of SE-encoding genes (*se* genes) in isolated strains represents a complementary approach for investigating SFPOs. All known *se* genes are located on mobile genetic elements, including the νSaβ genomic island which contains the enterotoxin gene cluster known as *egc* (carrying *seg* and *sei*), *S. aureus* pathogenicity islands (SaPIs; carrying *seb* and *sec*), prophages (carrying *sea*, *see*, and *sep*), and plasmids (carrying *sed*, *sej*, and *ser*; Argudin et al., 2012). Many PCR assays have been developed to detect *se* genes in *S. aureus* strains isolated from contaminated foods (Martin et al., 2004; Morandi et al., 2007; Kadariya et al., 2014). Screening for *se* genes in the strains involved in SFPOs is useful in two ways. First, the identified *se* gene may correspond to the type of SE detected in food, thus confirming the result obtained by an immuno-enzymatic method (Ostyn et al., 2010). Second, the *se* gene identified may correspond to a type of SE known to be emetic, but for which no detection method is available, suggesting the involvement of the corresponding toxin in the outbreak (Kerouanton et al., 2007).

The ANSES Laboratory for Food Safety is the French NRL and the EURL for CPS, including *S. aureus* and their toxins. One of the EURL activities is to develop and evaluate new molecular methods for bacterial typing and to transfer them to the European NRL network. Simultaneously to the screening for enterotoxins in suspected food, staphylococcal isolates are characterized using (i) spa-typing (ii) PFGE and (iii) a multiplex PCR assay for the detection of *se* genes coding for 11 SEs. Given the limitations described above, there is still a need for an alternative typing method that would be as discriminatory as PFGE and as portable as *spa*-typing, at a low cost.

Multiple-locus variable-number tandem-repeat analysis (MLVA) is based on PCR amplification and size analysis of DNA regions containing variable numbers of tandem repeats (VNTRs). MLVA assays offer fast typing of various bacteria, with high resolution (Lindstedt et al., 2013). An assay based on eight VNTR loci was applied on a panel of (i) 1781 *S. aureus* strains isolated from animal and patients (Schouls et al., 2009) and (ii) 78 strains related to SFPOs, in China, between 2010 and 2012 (Lv et al., 2014). Another MLVA assay targeting 14 loci was used in a survey of 309 strains including clinical methicillin-resistant *S. aureus* (MRSA) isolates and nasal carriage staphylococcal isolates (Pourcel et al., 2009). Finally, Sobral et al. (2012) proposed a third MLVA protocol based on the detection of 16 VNTR loci, including eight from Schouls et al. (2009) and eight from Pourcel et al. (2009). This protocol was implemented for the characterization of a panel (i) of 251 strains isolated primarily from humans and animals and also, to a lesser extent, from food and food poisoning samples (Sobral et al., 2012) and (ii) of 152 strains isolated from cases of bovine, ovine and caprine mastitits in France (Bergonier et al., 2014).

The aim of this study was to analyze the genetic diversity of a panel of *S. aureus* strains associated with SFPOs that occurred in France over the past 30 years. More specifically, we assessed the diversity of strains implicated in each outbreak and compared strains obtained from distinct outbreaks. MLVA data generated using the recent protocol of Sobral et al. (2012) were compared with those obtained by PFGE, spa-typing, and *se* gene detection. In light of our results, we discuss the usefulness of MLVA for routine typing of *S. aureus*, in terms of discriminatory power, and for investigating SFPOs.

## Materials and Methods

### Description of the Molecular Database

The French NRL has established a large collection of strains isolated from the main food production sectors throughout various French regions, over the past 30 years. This collection also includes clinical strains mainly obtained during collaborative research projects. All the strains have been typed by spa-typing and PFGE and characterized with regard to their *se* genes. The NRL molecular typing database (BioNumerics software, V 7.1, Applied Maths, Sint-Martens-Latem, Belgium) centralizes the epidemiological information, genotype and phenotype data for all the strains.

### Strain Panel

A panel of 112 strains isolated from 76 distinct SFPOs that occurred in France from 1981 to 2009 was selected for this study (**Table 1**). Out of these 112 strains, 13 strains were considered as epidemiologically related because they originated from four distinct “strong evidence” SFPOs (no “3,” “8,” “20,” “102”; **Table 2**). The epidemiological data regarding these four SFPOs were collected by the local health authorities using interviews or questionnaires. At the same time, tracing back of incriminated food was performed by the local services of the French Ministry in charge of agriculture and food. Three SFPOs (“3,” “8,” “20”) included food and clinical strains isolated within one region. The fourth SFPO (“102”) included only food strains isolated from three different regions in France. For this latter SFPO, a soft cheese made from unpasteurised cow’s milk was identified as the common and single source (Ostyn et al., 2010).

**Table 1:** Description of the 76 SFPOs that occurred in France from 1981 to 2009.

SFPO no.	Year	Incriminated food	Number of cases	SE detected in food	Number of strains analyzed per SFPO	SFPO assessment	SFPO reference in Kerouanton et al. (2007)
3	1999	Chocolate milk	NA^a^	SEA	3	Strong evidence	18
4	2000	Raw sheeps’ milk cheese	NA	SEA	1	Weak evidence	20
5	1985	Soft cheese	NA	SEA, SEB, SED	1	Weak evidence	7
6	1985	Soft cheese	NA	SEB	1	Weak evidence	8
7	1989	Chicken	NA	SEC	1	Weak evidence	12
8	2000	Sliced pork	NA	SEA, SED	3	Strong evidence	21
9	1981	Fresh cheese	NA	NT	2	Weak evidence	
10	1981	Raw milk semi-soft cheese	NA	SEA, SED	1	Weak evidence	1
11	2000	Soft cheese	NA	NT^b^	1	Weak evidence	
12	1986	Sheeps’ cheese	NA	NT	1	Weak evidence	
15	1994	Lasagna	NA	NT	1	Weak evidence	
16	1987	Unknown	NA	NT	1	Weak evidence	
17	1987	Milk	NA	NT	1	Weak evidence	
18	1992	Potato and rice salad	NA	SEA	1	Weak evidence	13
19	2001	Minced lamb meat	NA	NT	1	Weak evidence	
20	2000	Mixed salad	NA	SEC	3	Strong evidence	19
21	2001	Sliced soft cheese	NA	NT	1	Weak evidence	27
23	2001	Roasted pork	NA	SED	3	Weak evidence	28
24	2001	Leg of lamb	NA	SEA	1	Weak evidence	26
25	1983	Raw milk soft cheese	NA	ND^c^	1	Weak evidence	5
26	1983	Meat	NA	SEA, SED	1	Weak evidence	6
28	1987	Cake	NA	SEA	1	Weak evidence	10
29	1987	Strawberry tart	NA	NT	1	Weak evidence	
32	1983	Cooked beef	NA	SEA	1	Weak evidence	2
33	1983	Raw milk semi-soft cheese	NA	SEA, SED	1	Weak evidence	3
35	2001	Cream	NA	SEA	1	Weak evidence	25
36	1997	Nougatine	NA	SEA	1	Weak evidence	14
37	1988	Spaghetti	NA	SEA	1	Weak evidence	11
39	2001	Pancakes	NA	SEA, SED	2	Weak evidence	23
40	2001	Chocolate cake	NA	SEA, SED	1	Weak evidence	24
41	2001	Cooked rice	NA	NT	1	Weak evidence	
42	2001	Raw milk semi-soft cheese	NA	ND	4	Weak evidence	29
43	1997	Raw milk cheese	NA	SEA, SED	2	Weak evidence	15
45	1998	Raw milk semi-soft cheese	NA	ND	1	Weak evidence	17
46	2002	Raw sheep milk cheese	NA	SEA	1	Weak evidence	30
47	2002	Potted meat	NA	SEA	2	Weak evidence	31
49	2002	Cheese	NA	SEA	2	Weak evidence	
50	2002	Raw ham smoked	4	ND	1	Weak evidence	
52	2003	Custard topped with caramelized sugar	20	SEA, SED	1	Weak evidence	
53	2002	Custard topped with caramelized sugar	NA	NT	1	Weak evidence	
55	2003	Hard cheese made from raw milk	NA	NT	3	Weak evidence	
56	2004	Semi-solft cheese made from raw milk	5	ND	1	Weak evidence	
57	2004	Cheese	NA	SEA^d^	1	Weak evidence	
58	2004	Hard cheese made with raw milk	3	ND	1	Weak evidence	
59	2005	Strawberry shortcake	NA	SEA	1	Weak evidence	
60	2005	Potato salad	NA	SEA	2	Weak evidence	
61	2005	Chicken drumstick; Chicken drumstick Indian style	> 2	SEA	2	Weak evidence	
62	2005	Cake	NA	SEA	2	Weak evidence	
63	2005	Mussels and shrimp	NA	SEA	1	Weak evidence	
64	2006	Cheese	4	ND	3	Weak evidence	
65	2006	Coconut pastry	11	SEA, SED	2	Weak evidence	
66	2006	Smoked dry sausage	NA	ND	1	Weak evidence	
69	2007	Corned beef hash, cottage pie	3	ND	1	Weak evidence	
70	2007	Sauerkraut	3	NT	1	Weak evidence	
71	2007	Meat raviolis	NA	NT	1	Weak evidence	
72	2007	Minced chicken vetetable mix for sandwich	10	SEA, SEC	1	Weak evidence	
73	2007	Semolina, vegetables and sweet red peper; pepper and vegetables	22	SEA	2	Weak evidence	
80	2007	Pasta	NA	NT	1	Weak evidence	
81	2007	Semi-solft cheese	NA	NT	2	Weak evidence	
82	2007	Cheese made from raw milk	NA	ND	1	Weak evidence	
83	2007	Coconut pastry frozen	3	SEA	3	Weak evidence	
84	2007	Goat milk	NA	NT	2	Weak evidence	
85	2007	Mixed salad	20	SEA, SEC	1	Weak evidence	
86	2008	Duck liver	NA	NT	1	Weak evidence	
87	2008	Soft cheese	5	SED	4	Weak evidence	
88	2008	Ham hock leftovers	2	ND	1	Weak evidence	
91	2008	Pasta salad	100	ND	1	Weak evidence	
92	2008	Baked salmon in foil	47	SEA	2	Weak evidence	
94	2008	Tuna, mayonnaise, salad	15	SEA	1	Weak evidence	
96	2009	Ripened sheeps’ cheese made with raw milk	27	ND	1	Weak evidence	
97	2009	Chicken filet	10	SEA	1	Weak evidence	
98	2009	Fruit pie with cream	4	SEA	1	Weak evidence	
99	2009	Mackerel mustard sauce	21	SEA, SEC	1	Weak evidence	
100	2009	Boned ham	3	SEA	1	Weak evidence	
101	2009	Spare rib smoked	NA	NT	1	Weak evidence	
102^e^	2009	Soft cheese	23	SEE	4	Strong evidence	

*^a^NA, not available; ^b^NT, not tested; ND, not detected (Se detected at a concentration inferior to 20 ng/ml); ^c^ND, not detected; ^d^SEs were detected at a concentration of between 20 and 100 ng/ml; ^e^(Ostyn et al., 2010 #299).*

**Table 2:** Typing data on the 13 strains related to four distinct “strong evidence” SFPOs.

SFPO no.	Strain no.	Strain origin	Food product and batch number	*Spa* type	SmaI-pulsotype	PCR *se* genes	MLVA profile
3	255D	Clinical	Chocolate milk	t127	21	*sea, seh*	11
	256D	Clinical		t127	21	*sea, seh*	11
	257D	Food		t127	21	*sea, seh*	11
8	355E	Clinical	Pork ribs	t008	91	*sea, sed, sej*	43
	354E	Clinical		t008	91	*sea, sed, sej*	43
	349E	Food		t008	91	*sea, sed, sej*	43
20	377F	Clinical	Mixed salad	t156	44	*sec*	79
	378F	Clinical		t156	44	*sec*	79
	293E	Food		t156	44	*sec*	79
102	09CEB319STA	Food	Soft cheese 3412	t4461	155	*se*	55
	09CEB319STA	Food	Soft cheese 3402	t4461	155	*se*	55
	09CEB314STA	Food	Soft cheese 3403	t4461	155	*se*	55
	09CEB329STA	Food	Soft cheese 3405	t4461	155	*se*	55

The 99 remaining strains were isolated from 72 different “weak evidence” SFPOs that occurred in different locations in France between 1981 and 2009 (**Table 1**). Out of these 72 “weak evidence” SFPOs, 53 involved only one strain, and the remaining 19 involved between two and four strains isolated from different food samples (**Table 3**).

**Table 3:** Multiple-locus variable-number tandem-repeat analysis (MLVA) and pulsed field gel electrophoresis (PFGE) typing data for the 46 strains originating from the 19 “weak evidence” SFPOs including several strains.

SFPO no.	Number of strains	Total number of distinct MLVA types	Total number of distinct PFGE types
42	4	4	2
87	4	4	4
23	3	1	1
55	3	2	2
83	3	3	3
64	3	2	1
81	2	2	2
65	2	1	1
49	2	2	1
62	2	2	1
47	2	1	1
73	2	1	1
61	2	1	1
84	2	2	2
60	2	2	2
43	2	1	1
9	2	2	2
92	2	2	2
39	2	2	2
**Total**	46	37	32

To compare the discriminatory power of spa-typing, MLVA and PFGE, 43 additional strains were included in the panel. These 43 strains were selected according to their PFGE profiles to represent the genetic diversity observed in the molecular database. Then, we selected a total panel of 146 epidemiogically unrelated isolates comprising one strain of each of the four “strong evidence” SFPO (i.e., four isolates), 99 strains related to each of the “weak evidence” SFPOs (99 isolates), four strains related to four outbreaks that occurred in Belgium, one strain related to one outbreak in Japan (Omoe et al., 2003; Ono et al., 2008), 31 strains isolated from research projects and seven strains isolated from monitoring programs. This panel included food strains (*n* = 122 with 112 isolated from SFPOs) and strains isolated from either human cases (*n* = 19) or animals (*n* = 5).

### DNA Extraction

All strains were stored in cryobeads at -80°C. They were cultured overnight at 37°C in brain heart infusion (BHI), isolated on a non-selective medium (Milk Plate Count Agar) and incubated at 37°C for 24 h, prior to extraction of total DNA. DNA extraction was performed using the InstaGene kit (Bio-Rad, Marnes-la-Coquette, France) according to the manufacturer’s recommendations. DNA concentrations were adjusted approximately to 100 ng/μl using a Nanodrop1000 spectrophotometer (spectrophotometer, Thermo scientific, Wilmington, DE, USA).

### Detection of *se* Genes Using the EURL Multiplex PCR Assay

Two multiplex PCR systems were used to detect the genes of 11 types of enterotoxins, i.e., *sea*, *seb*, *sec*, *sed*, *see*, *seg*, *seh*, *sei*, *sej*, *sep*, and *ser*. The primers used to amplify the *sea*, *seb*, *sec*, and *sed* genes were designed by Mehrotra et al. (2000), those for *seg*, *seh*, *sei, sej*, and *sep* by Bania et al. (2006), those for *see* by Sharma et al. (2000), and those for *ser* by Chiang et al. (2008). They were synthesized by Eurofins (MWG Operon, France).

For the multiplex reaction targeting *sea*, *seb*, *sec*, *sed*, *see*, and *ser* genes, the 25 μl reaction mixture contained 1 U Fast Start Taq DNA polymerase (Roche, Diagnostics, Meylan, France), 2.5 mM MgCl_2_, 0.2 mM dNTPs, 1X PCR buffer, 0.2 μM of each primer for *sea*, *seb*, *sec*, *ser*, 0.8 μM of each primer for *sed*, 0.6 μM of each primer for *see* and 2 μl of DNA. PCR was performed on a Veriti^®^ PCR cycler (Applied Biosystems, Courtaboeuf, France). The thermal cycle included an initial denaturation at 94°C for 3 min, followed by 35 cycles of denaturation at 94°C for 30 s, annealing at 56°C for 40 s, extension at 72°C for 1 min 30, and a final extension at 72°C for 7 min. The conditions for the multiplex targeting *seg*, *seh*, *sei*, *sej*, and *sep* genes were as described above, except that the annealing step was performed at 53°C for 40 s and the reaction mixture included 0.8 μM of each primer for *seg*, *sei*, *sej*, *sep* and 0.4 μM of each primer for *seh*. DNA of each isolate was tested by polymerase chain reaction (PCR) targeting the ribosomal RNA 23S gene region specific for *S. aureus* (Kerouanton et al., 2007).

Five reference *S. aureus* strains (i.e., FRIS6, 374F, FRI137, HMPL280, FRI326) were used as positive controls. The PCR products were separated by electrophoresis in a 2% agarose gel and visualized using the Gel Doc EQ apparatus (Bio-Rad).

### MLVA Typing

DNA samples were diluted in molecular grade water to obtain solutions at 10 ng/μl. They were used as DNA templates for PCR amplification according to the protocol described by Sobral et al. (2012) with minor modifications to the amplification program: DNA template concentration was set at 10 ng/μl (instead of 5 ng/μl in Sobral’s protocole), touchdown PCR and long range PCR were both set at 18 thermal cycles (instead of 15). Samples were loaded onto an ABI3500^®^ capillary sequencer using a 50 cm capillary filled with performance-optimized polymer 7 (Applied Biosystems) at 60°C for 6200 s with a running voltage of 12 kV, and an injection time and voltage of 10 s and 1.6 kV, respectively.

For each multiplex reaction, 2 μl of purified PCR product was combined with 7.75 μl HiDi formamide and 0.25 μl GS1200LIZ (Applied Biosystems). Samples were loaded onto an ABI3500^®^ capillary sequencer using a 50 cm capillary filled with performance-optimized polymer 7 (Applied Biosystems) at 60°C for 6200 s with a running voltage of 12 kV, and an injection time and voltage of 10 s and 1.6 kV, respectively.

Each run included a negative (water) control to ensure the absence of contamination and a positive control to verify the PCR reaction.

From the panel of 112 strains, 12 strains related to “weak evidence” SFPOs (no 431G, 360F, 338E, 419G, 372F, 402F, 353E, 301E, 384F, 339E, 363F, 399F) that had previously been tested by MLVA by Sobral et al. (2012) were used here as positive controls.

Amplification products were electrophoresed twice in independent runs. At least two independent PCRs were performed from a given DNA extract of the reference strains.

#### Data Analysis

The products of both multiplex PCR amplifications were resolved by capillary electrophoresis, and the alleles from each of the 16 targeted loci were automatically identified. Each VNTR locus was identified according to specific fluorescent dyes and automatically assigned to a DNA fragment size by the GeneMapper software (Applied Biosystems). This size was then converted into an allele designation according to the number of repeats found on the fragment, in associated with a quality index. The typing data file was imported into the NRL molecular database.

Minimum spanning trees were constructed using a categorical coefficient and unweighted pair group method with arithmetic mean (UPGMA) clustering. Allele designations and nomenclature were used according to Sobral et al. (2012). Partial repeats were rounded down to the closest half decimal (e.g., 1.0, 1.5, 2.0).

#### Sequence Verification

Any new alleles of unexpected size were sequenced. The loci and flanking regions were amplified in both directions with high-fidelity HotStart Taq Polymerase (Roche Diagnostics). Amplification products were sequenced by Eurofins (MWG Operon, France). The sequence analysis was performed into the NRL molecular typing database.

### PFGE Typing

The initial step of PFGE as defined by the EURL for CPS protocol was performed according to the protocol described by Chung et al. (2000) with minor modifications. Briefly, strains were cultured in liquid BHI (instead of liquid TSB medium). Cell density was determined from a 2 ml suspension (instead of 0.5 ml) at 600 nm (instead of 620 nm). The agarose plugs were prepared in TE buffer (instead of PIV) and the PIV buffer contained 2 mM Tris-HCL and 1 M NaCl (instead of 10 mM Tris, 1 M NaCl). Plug shape was cubic (instead of circular) and EC buffer was incubated for 3 h (instead of 5 h).

The protocol for the subsequent steps of PFGE was based on the recommendations of the Harmony typing group (Murchan et al., 2003). The reference standard, *S. aureus* NCTC 8325 *Sma*I profile, was loaded in every fifth or sixth lane. The total running time was 20 h, the first-block switch time was 5–15 s for 8.5 h, and the second-block switch time was 15–60 s for 11.5 h. The voltage applied for the run was 6 V/cm. The CHEF DRIII system (Bio-Rad) was used, with an included angle of 120° and a linear ramp factor.

Gels were stained for 30 min in a 400 ml ultra- pure sterile water solution containing ethidium bromide at 10 mg/ml and banding profiles were visualized under UV light, using the Gel Doc Eq system and Quantity One software (Bio-Rad). DNA profiles were analyzed in the NRL molecular typing database. PFGE pulsotypes were considered as different if there was at least one band different between them (Barrett et al., 2006). Each PFGE profile was arbitrarily assigned to a pulsotype number.

### Spa-Typing

*Spa*-typing was performed as previously described by Shopsin et al. (1999) and Aires-de-Sousa et al. (2006).

A strain already known for its *spa*-type (*S. aureus* Mu50, spa-type t002, Kuroda et al., 2001) was used as a positive control and a reaction without DNA was included within each run as a negative control. The PCR products were migrated on a 2% agarose gel and visualized using the Gel Doc EQ apparatus (Bio-Rad). They were sequenced by Eurofins (Esberg, Germany), on both DNA strands. The sequences were analyzed using BioNumerics software which provides a fully automated workflow, from import of raw sequencer trace files to assignment of repeat codes and *spa* types using the plug-in *spa*-typing which connects to SeqNet/Ridom Spa Server^1^. Each new base composition of the polymorphic repeat found in a strain was assigned a unique repeat code. The succession of repeats in a given strain determines the strain’s *spa* type. New *spa*-types were submitted to the SeqNet server^2^.

### Epidemiological Concordance, Discriminatory Power, and Congruence of the Typing Methods

The epidemiological concordance of PFGE, *spa*-typing and MLVA was assessed by testing their capacity to recognize the homogeneity of 13 strains related to four distinct “strong evidence” SFPOs (**Table 2**) in the same epidemiological groups.

The ability of the methods to discriminate *S. aureus* strains (i.e., unrelated strains) was assessed by calculating Simpson’s index of diversity (ID; Hunter and Gaston, 1988) with confidence interval calculated according to (Carriço et al., 2006). The ID was calculated from PFGE, spa-typing and MLVA results obtained from the panel of 146 epidemiologically unrelated isolates.

This strain panel was reduced of four strains not typable by spa-typing, and used for congruence test between spa-typing, MLVA and PFGE. The congruence assessments were performed using the adjusted Rand’s coefficient (Carriço et al., 2006, #1093). Adjusted rand coefficient consider (i) the probability that a pair of isolates which is assigned to the same type by one typing method is also typed as identical by the other method, (ii) the probability that a pair of isolates which is assigned to two types by one typing method is also typed as different by the other method and corrects the typing concordance for chance agreement, avoiding the overestimation of congruence between typing methods.

For a finer comparison the adjusted Wallace (AW) coefficients (Severiano et al., 2011, #1095) were also performed. Statistics analysis were performed in BioNumerics software, V 7.1, (Applied Maths) using a script developed by Ana Severiano and João André Carriço available online at http://darwin.phyloviz.net/ComparingPartitions/. The AW coefficient indicates the probability that pairs of isolates which are assigned to the same type by one typing method are also typed as identical by the other and corrects the typing concordance for chance agreement. The AW coefficient is directional, i.e., given a standard method. It considers the probability of two strains having the same type of standard method also sharing the same type of the compared method.

## Results

### Discriminatory Power and Concordance of MLVA, PFGE, and Spa-Typing

All the strains were identified as *S. aureus* by the 23S rDNA PCR assay specific for this bacterial species. The ID diversity index was assessed on a panel of 146 epidemiologically unrelated strains, which included 103 strains associated with various French SFPOs. MLVA and PFGE separated the 146 strains into 125 and 118 distinct groups, respectively. *Spa*-typing separated 142 out of the 146 strains into 71 groups only (**Table 4**). The four remaining strains could not be typed using this technique. High ID values were observed for MLVA (0.997) and PFGE (0.995) indicating that almost each strain can be distinguished from all other members of the strain panel by these two typing methods. PFGE and MLVA were found to be more discriminatory than *spa*-typing whose ID value was 0.970 (**Table 4**).

**Table 4:** Multiple-locus variable-number tandem-repeat analysis, PFGE and spa-typing results from a panel of 146 not epidemiologically related strains.

	Number of analyzed strains	Number of successfully typed strains	ID and CI 95%	Total number of distinct types	Total number of unique types
MLVA	146	146	0.997 (0.995–0.999)	125	109
PFGE	146	146	0.995 (0.992–0.998)	118	100
*spa*-typing	146	142	0.970 (0.959–0.982)	71	50

*ID, Simpson’s index of diversity (Hunter and Gaston, 1988, #1092).*

*CI, confidence interval.*

Four PFGE groups and five MLVA groups were shared mainly into two different spa-types. Eight MLVA groups were shared mainly into two PFGE types. Eleven PFGE groups were shared mainly into two MLVA types (data not shown).

Quantitative determination of concordance of the three methods was calculated (**Table 5**). Congruence between the three methods was found to be low with adjusted Rand coefficient values ranging from 0.131 to 0.269 (**Table 5**). The AWMLVA→spa-typing and AWPFGE→spa-typing were respectively higher than AWspa-typing→MLVA and AWspa-typing→PFGE (**Table 5**) indicating that partitions defined by spa-typing could have been predicted from the results of MLVA or PFGE.

**Table 5:** Congruence between the typing methods using adjusted Rand and adjusted Wallace coefficients.

Method	Adjusted Rand coefficient			Adjusted Wallace coefficient 95% CI		
	Spa-typing	MLVA	PFGE	Spa-typing	MLVA	PFGE
spa-typing					0.072	0.104
					(0.021–0.123)	(0.043–0.166)
MLVA	0.131			0.725		0.363
				(0.621–0.830)		(0.212–0.515)
PFGE	0.178	0.269		0.616	0.213	
				(0.429–0.804)	(0.085–0.342)	

*CI, confidence interval.*

### Typing of 112 French SFPO Strains

#### Typing Data from Four “Strong Evidence” SFPOs (13 Strains)

Typing of 13 *S. aureus* strains from four “strong evidence” SFPOs showed that the *se* gene profiles and the MLVA-, PFGE-, and spa- types were indistinguishable within each set of epidemiologically related strains (**Table 2**). This demonstrated the good epidemiological concordance of the four methods.

#### Typing Data from 76 “Weak Evidence” SFPOs (103 Strains)

##### MLVA

A panel of 103 epidemiologically unrelated strains from 76 French SFPOs was selected, containing one strain of each of the four “strong evidence” SFPOs described above (**Table 2**) and 99 strains from 72 “weak evidence” SFPOs (**Table 1**). For 12 strains previously tested elsewhere (i.e., 431G, 360F, 338E, 419G, 372F, 402F, 353E, 301E, 384F, 339E, 363F, 399F), the MLVA profiles obtained here were similar to those obtained by Sobral et al. (2012).

Multiple-locus variable-number tandem-repeat analysis separated the 103 strains into 84 different types. The most prevalent MLVA types were “20” and “1,” with each of these two types containing four strains isolated from four different SFPOs that occurred over the periods 1983–2007 and 2000–2008, respectively. The incriminated food was different for each of these SFPOs.

##### PFGE compared with MLVA

Pulsed field gel electrophoresis separated the 103 strains into 80 pulsotypes. The most prevalent PFGE type was “18” and contained five strains isolated from four distinct SFPOs that occurred between 1999 and 2008.

Out of the 76 different “weak evidence” SFPOs, 19 included several strains (i.e., between two and four strains; **Table 3**). For 15 of these SFPOs, strains that displayed distinct MLVA profiles also displayed distinct PFGE pulsotypes, and strains with similar MLVA profiles also showed similar PFGE profiles. MLVA data were therefore in agreement with those of PFGE. For three of the 19 SFPOs (i.e., “62,” “49,” “64”), the strains had different MLVA profiles but showed indistinguishable PFGE pulsotypes (**Table 3**). However, for the SFPOs “49” and “64,” the MLVA profiles obtained were very similar, differing by only 1.5 repeat. For the remaining SFPO (i.e., “42”), the four strains displayed four different MLVA profiles but showed only two distinct PFGE pulsotypes (**Table 3**).

##### Spa-typing compared with MLVA

**FIGURE 1:**
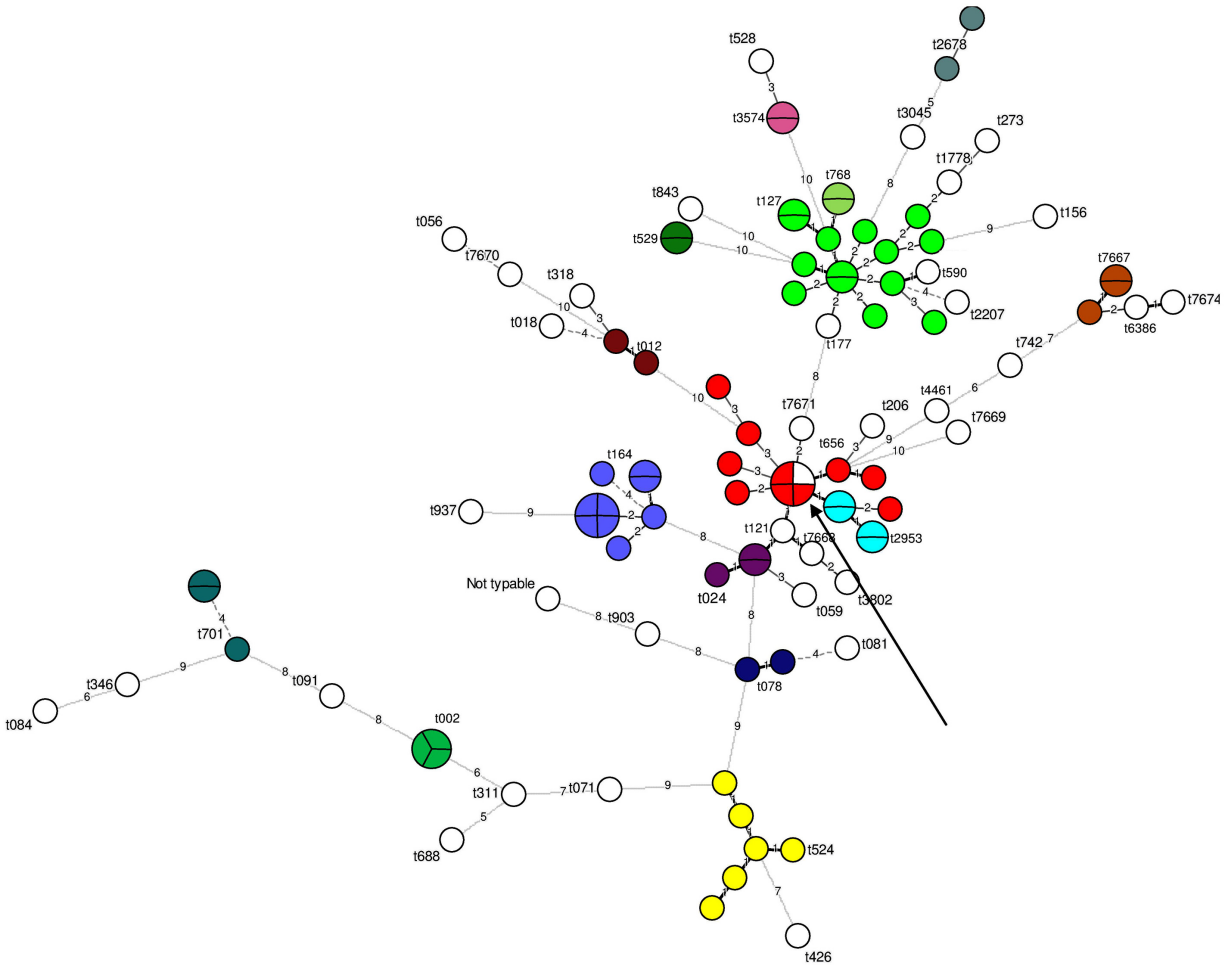
**Minimum spanning tree of spa-types according to the multiple-locus variable-number tandem-repeat analysis (MLVA) types determined on the 103 strains tested.** 102 strains were successfully typed using *spa*-typing. Each circle represents a particular MLVA type. The size of each circle is proportional to the number of isolates within the MLVA type. The distance between the circles represents the genetic divergence. The divergence is given in number of mutations and is indicated on the branch. Each color represents a different spa-type (*n* > 1 isolate). All unique (*n* = 1 isolate) *spa*-type are shown in white. The arrow shows the single MLVA type including two different spa-types.

*Spa*-typing separated 102 out of the 103 strains into 50 different types; one strain could not be typed. The most frequent *spa*-types observed were t127 (*n* = 14) and t008 (*n* = 10; **Figure 1**). Five new *spa*-types were observed for the first time here (i.e., t7667, t7668, t7669, t7671, and t7674). Except for one MLVA type (“20”), all the MLVA types that included several strains corresponded to the same spa-type. The type”20” included one and three strains with two different spa-types, t656 and t008, respectively (**Figure 1**). These two *spa-*types were close: they had the same repeat number and only differed at two single nucleotide positions in the second *spa* repeat.

##### PCR *se* genes compared with MLVA

Out of the 103 strains tested, 16 strains did not carry any *se* gene and 87 strains carried at least one *se* gene. These 87 strains were divided into 20 distinct *se* gene profiles. The most frequently occurring gene was *sea* (*n* = 58), followed by *seg* (*n* = 23) and *sei* (*n* = 23), by *seh* (*n* = 19) and then by *sed*, *sej*, *ser*, *sec*, *sep*, *seb*, and *see*. Several *se* genes could be present, and the most frequent associations of *se* genes detected were *sea-seh* (*n* = 15), *sea-sed-sej-ser* (*n* = 13) and *seg-sei* (*n* = 11). Moreover, 65 strains carried genes corresponding to ‘new’ enterotoxins, i.e., *seg*, *seh*, *sei*, *sej*, *sep*, and *ser.* Eleven strains carried the *seg* and *sei* genes and one strain the *seg*, *sei*, *sej*, *sep*, and *ser* genes.

**FIGURE 2:**
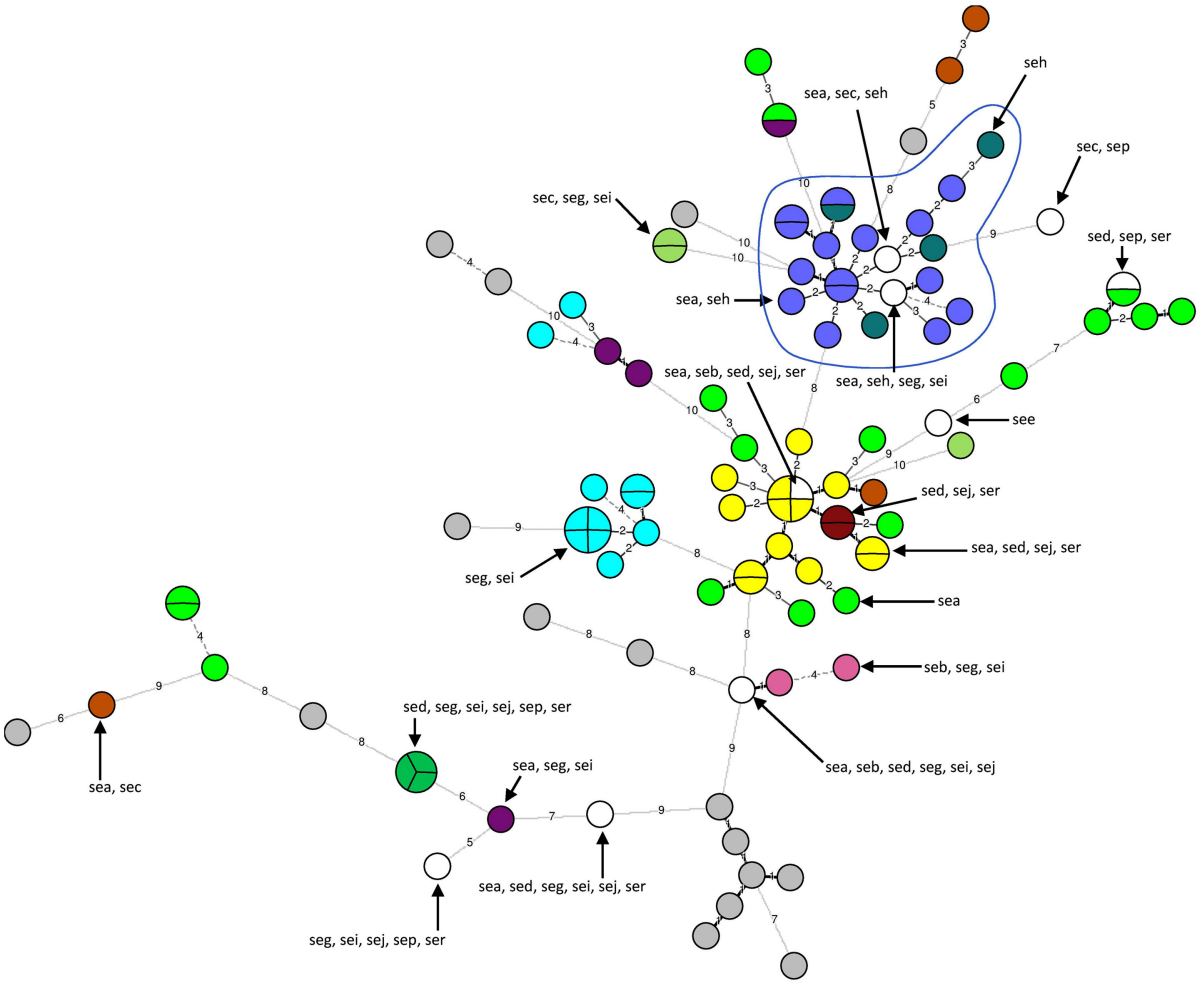
**Minimum spanning tree of 103 *Staphylococcus aureus se* gene-carrying strains according to their MLVA types. Each circle represents a particular MLVA type**. The size of each circle is proportional to the number of isolates within this MLVA type. The distance between the circles represents the genetic divergence. The divergence is given in number of mutations and is indicated on the branch. Each color represents a particular *se* gene combination (*n* > 1 isolate), with unique *se* combination (*n* = 1 isolate) shown in white and no *se* gene shown in gray. Four MLVA-types had strains with different *se* genes combinations (circles with several colors). The MLVA subgroup that includes seh-carrying strains is highlighted in blue and circled. A subgroup groups strains with MLVA types that differ by a maximum of five mutations.

The 16 strains for which no *se* gene were detected were divided into 16 unique MLVA types. The 87 *se* gene-carrying strains were separated into 68 distinct MLVA-types: 64 MLVA-types correlated with *se* gene carriage (**Figure 2**). The four remaining MLVA-types included strains with different *se* genes as indicated by circles including various colors in the **Figure 2**. The 21 strains that carried *seh* either alone or in combination with *sea* were divided into 19 very close MLVA types that clustered within the same MLVA subgroup (**Figure 2**). 14 out of the 21 *seh*-carrying strains that clustered within the same MLVA sub-group possessed the spa type t127 (**Figures 1** and **2**).

## Discussion

### Comparison MLVA with PFGE

The aim of this study was to investigate the genetic diversity of 112 *S. aureus* strains involved in SFPOs in France between 1981 and 2009. The MLVA protocol developed by Sobral et al. (2012) was compared here for the first time with the EURL PFGE protocol. The analysis of a panel of 146 *S. aureus* strains isolated from different sources (clinical cases, food, animal cases, or SFPOs) showed that MLVA and PFGE have equal discriminatory power.

For the strains related to the strong evidence SFPOs, MLVA, and PFGE typing data demonstrated the good epidemiological concordance of each of the two methods. Moreover, for the strains which were involved in the same “weak-evidence” SFPO MLVA could discriminate strains which had similar PFGE types.

With the MLVA protocol used here, results could be obtained within 48 h, i.e., faster than PFGE analysis which is usually completed in 3 days from receipt of pure culture. Moreover, the method is easy to perform, is readily automatable, and allows high sample throughput. The use of commercially available reagents can help foster standardization. MLVA data are also suitable for electronic transmission between laboratories and are not prone to subjective interpretation. Moreover, this MLVA method benefits from the higher resolution of using a capillary electrophoresis methodology over standard gel-based techniques. The method can therefore be easily implemented by NRLs equipped with a capillary electrophoresis system. One inconvenience could be the use of an expensive system. Nevertheless, this MLVA protocol is also suitable for agarose-gel electrophoresis. Indeed, for each of the 16 VNTRs, repeat size was sufficiently high for accurate band sizing on agarose gels. High-throughput typing on agarose gels can also be facilitated by the use of an automated flow-through gel electrophoresis system (Reskova et al., 2014).

The major drawback of this MLVA assay is the lack of free internet-accessible databases for comparative purposes. To date, the databases used worldwide^3^ (MLVA.net) centralize profiles obtained with the protocols of Pourcel et al. (2009) and Schouls et al. (2009) using subset of loci. However, the development of an easily accessible database centralizing profiles obtained from the 16 VNTRs with this protocol is in progress and should be available in the coming years.

### Prevalence of *se* Genes among the SFPO Strains

In this study, 65 out of 87 enterotoxigenic strains carried more than one gene coding for enterotoxins, illustrating the importance of searching for *se* genes in the strains involved in SFPOs. Our results confirmed the clear predominance of the *sea* gene (67%) among the SFPO strains and its frequent association with other genes such as *seh* or *sed-sej-ser*. The prevalence of the *sea* gene in the strains linked to SPFOs has been already observed in Spain (Dyer et al., 2007; Sobral et al., 2007; Kellermann et al., 2008), Italy (Morandi et al., 2007), and in Japan (Sato’o et al., 2014). The association of *sea* with *seh* has also been observed for strains linked to SFPOs in France (Kerouanton et al., 2007) and recently in Japan (Sato’o et al., 2014).

### Genetic Diversity and Origin of Strains Involved in the French SFPOs

The three typing method, MLVA, PFGE and spa-typing, show ID greater than 0.90. This value is considered as a cut-off in order to interpret the typing results with confidence (Hunter and Gaston, 1988, #1092; Matallah et al., 2013, #1096). PFGE and MLVA bring more information than spa-typing.

Whatever the typing method used here for the analysis of 112 French SFPOs-isolates, i.e., MLVA, PFGE, spa-typing or *se* genes detection, a large number of different molecular types was found, highlighting the high genetic diversity of the tested isolates. In this study, associations between MLVA types, *se* gene combinations and spa-types were identified. All the 21 *seh*-carrying strains clustered within the same MLVA sub-group, and 14 of these showed spa type t127. These results demonstrated that the strains carrying the *seh* gene have a genetically related background, as previously suggested by Ruzickova et al. (2008) for 28 enterotoxin H-positive *S. aureus* strains isolated from food samples in the Czech Republic.

Previously, a high concordance between the MLVA protocol and the MLST method was demonstrated and a distribution signature of clonal complexes typical of human isolates was found for 13 SFPO strains (Sobral et al., 2012). Further comparison of the MLVA data obtained here from 112 SFPO strains with those from a representative panel of human strains could be useful to confirm the hypothesis of a human origin for SFPO strains.

Another investigation could be useful to explore the genetic structure of all the populations of food *S. aureus* strains associated to SFPO isolated in France over the past 30° years.

## Conclusion

By combining various molecular typing methods, we highlighted the high genetic diversity of *S. aureus* strains involved in SFPO in France over the past 30 years. In addition, the MLVA protocol developed by Sobral et al. (2012) was found highly discriminatory, therefore representing a very interesting alternative to PFGE for establishing epidemiological links during SFPOs investigations. This MLVA method could be used also to better understand the population biology of *S. aureus*. Before transferring this MLVA protocol through the European NRL network, its reproducibility will need to be assessed in proficiency testing trials, in order to compare and interpret MLVA data and harmonize assignment of MLVA types.

## Author Contributions

SR participated in the design and coordination of the study, the data interpretation and the draft of all the manuscript. BF participated to the design of the study, the data interpretation under BioNumerics software and the strain typing by PFGE, spa-typing and *se* genes. NV was in charge of the strain typing by MLVA and MLVA data analysis. JG carried out all the PCR tests at EURL. J-AH took part to the draft of the manuscript. LG carried all the congruence analysis and took part to the draft of the manuscript. AB and FA participated in the design and coordination of the study, and the draft of all the manuscript. All authors read and approved the final manuscript.

## Conflict of Interest Statement

The authors declare that the research was conducted in the absence of any commercial or financial relationships that could be construed as a potential conflict of interest.

## Footnotes

1 http://spaserver.ridom.de/

2 http://www.seqnet.org/spaserver.shtml

3 http://minisatellites.u-psud.fr

## Abbreviations

CPS: coagulase-positive staphylococci
EURL: European Union Reference Laboratory
NRL: National Reference Laboratory
SEs: staphylococcal enterotoxins
SFPO: staphylococcal food poisoning outbreaks.
